# Electrochemically Inert Li_2_MnO_3_: The Key to Improving the Cycling Stability of Li-Rich Manganese Oxide Used in Lithium-Ion Batteries

**DOI:** 10.3390/ma14164751

**Published:** 2021-08-23

**Authors:** Lian-Bang Wang, He-Shan Hu, Wei Lin, Qing-Hong Xu, Jia-Dong Gong, Wen-Kui Chai, Chao-Qi Shen

**Affiliations:** State Key Laboratory Breeding Base of Green Chemistry-Synthesis Technology, College of Chemical Engineering, Zhejiang University of Technology, Hangzhou 310014, China; wanglb99@zjut.edu.cn (L.-B.W.); zjuthuheshan@163.com (H.-S.H.); 2112001474@zjut.edu.cn (W.L.); 2111901309@zjut.edu.cn (Q.-H.X.); 2111901065@zjut.edu.cn (J.-D.G.); kikiihac@gmail.com (W.-K.C.)

**Keywords:** lithium-rich manganese oxide, nanocomposite, dynamic hydrothermal, inert Li_2_MnO_3_, cycling stability

## Abstract

Lithium-rich manganese oxide is a promising candidate for the next-generation cathode material of lithium-ion batteries because of its low cost and high specific capacity. Herein, a series of xLi_2_MnO_3_·(1 − x)LiMnO_2_ nanocomposites were designed via an ingenious one-step dynamic hydrothermal route. A high concentration of alkaline solution, intense hydrothermal conditions, and stirring were used to obtain nanoparticles with a large surface area and uniform dispersity. The experimental results demonstrate that 0.072Li_2_MnO_3_·0.928LiMnO_2_ nanoparticles exhibit a desirable electrochemical performance and deliver a high capacity of 196.4 mAh g^−1^ at 0.1 C. This capacity was maintained at 190.5 mAh g^−1^ with a retention rate of 97.0% by the 50th cycle, which demonstrates the excellent cycling stability. Furthermore, XRD characterization of the cycled electrode indicates that the Li_2_MnO_3_ phase of the composite is inert, even under a high potential (4.8 V), which is in contrast with most previous reports of lithium-rich materials. The inertness of Li_2_MnO_3_ is attributed to its high crystallinity and few structural defects, which make it difficult to activate. Hence, the final products demonstrate a favorable electrochemical performance with appropriate proportions of two phases in the composite, as high contents of inert Li_2_MnO_3_ lower the capacity, while a sufficient structural stability cannot be achieved with low contents. The findings indicate that controlling the composition through a dynamic hydrothermal route is an effective strategy for developing a Mn-based cathode material for lithium-ion batteries.

## 1. Introduction

Since rechargeable lithium-ion batteries were first applied to electronic products in the 1990s, their development has been continual [1]. After three decades, Li-ion batteries have evolved and become an essential component of well-established energy storage strategies, with excellent efficiency in terms of energy and power densities, life span, and design flexibility [2,3]. Meanwhile, the storage demand from clean energy technologies requires Li-ion batteries, given their merits of low cost, high safety, and environmental compatibility [4]. Compared with new generation anode materials, such as silicon–carbon composites with a specific capacity of 700–2000 mAh g^−1^, improvement in the capacity of cathode materials is somewhat lagging [5,6]. As the bottleneck of capacity and energy density, cathode materials are believed to be the main factor when further optimizing the electrochemical performance and addressing other issues of Li-ion batteries [5,7].

Currently, the most widely used cathode materials are ternary NMC and LiFePO_4_, while Li-rich manganese-based materials have attracted considerable attention due to their low cost and high specific capacity. Generally, this type of cathode material, noted as xLi_2_MnO_3_·(1 − x)LiTMO_2_ (transition metal (TM) = Ni, Co, and Mn, etc.), exhibits a superior specific capacity (>250 mAh g^−1^) and high operation voltage to realize an excellent energy density; thus, xLi_2_MnO_3_·(1 − x)LiTMO_2_ is assumed to be a promising cathode material for the next generation of lithium-ion batteries [8]. However, the large price fluctuation of cobalt and nickel in recent years, as well as their negative impact on the environment, have driven researchers to design Li-rich manganese oxide without Ni and Co, using xLi_2_MnO_3_·(1 − x)LiMnO_2_ as a substitute [9,10]. The preparation routes of xLi_2_MnO_3_·(1 − x)LiMnO_2_ include solid-state calcination [11], sol–gel synthesis [12], pyrolysis reduction [13], and hydrothermal/solvothermal reaction [14]. The specific capacity of xLi_2_MnO_3_·(1 − x)LiMnO_2_ has been significantly improved in comparison with common lithium manganese oxides (LiMn_2_O_4_, LiMnO_2_), but its capacity degradation during cycling is relatively severe [15].

The structure of the xLi_2_MnO_3_ (1 − x)LiMnO_2_ composite is believed to be a mixture of the Li_2_MnO_3_ and LiMnO_2_ crystal domains [16,17], while the hypothesis of a solid-state solution has also been presented in some studies [18,19]. In the composite, the crystal structure of Li_2_MnO_3_ is combined with monoclinic C2/m, but LiMnO_2_ can display diverse structures, such as monoclinic or orthorhombic structures, when using different synthetic methods. In most publications, Li_2_MnO_3_ was activated in the initial cycles and provided an extra capacity, similarly to xLi_2_MnO_3_·(1 − x)LiTMO_2_ mentioned above. During the activation process, the conjoint removal of Li^+^ and O forms an active “MnO_2_-like” phase, and anionic and cationic vacancies are generated simultaneously to cause lattice densification [20,21]. Irreversible O_2_ extraction (Li_2_MnO_3_ → Li_x_MnO_2_ + (2 − x)Li^+^ + 1/2O_2_ + (2 − x)e^−^), occupation of the Li^+^ site by the transition metal, and phase transformation to spinel or rock salt induce a decline in capacity [22,23,24]. While recent research has demonstrated that Li_2_MnO_3_ is activated to generate oxygen anion O^n^**^−^** (n < 2), redox of the oxygen anion during cycling could contribute considerably to increasing its capacity [25]. In order to improve the stability of the material and to mitigate the reduction in capacity due to structure evolution, elemental doping and surface modification with oxides are widely applied [26]. However, applying additional optimization treatment procedures generally goes against attempts to develop cost-effective and time-saving methods for the production of cathode materials [27].

In this work, a xLi_2_MnO_3_·(1 − x)LiMnO_2_ composite was prepared via a one-step dynamic hydrothermal synthetic route; interestingly, the contained Li_2_MnO_3_ was not activated even when cycled under 2–4.8V for 15 cycles. The x-values of 0.045, 0.072, and 0.114 in xLi_2_MnO_3_·(1 − x)LiMnO_2_ were characterized by ICP–OES, and the sample containing 0.072 Li_2_MnO_3_ manifested the best electrochemical performance. The inertness of Li_2_MnO_3_ is ascribed to its high crystallinity with few defects, as its presence effectively improved the electrochemical cycling capability of xLi_2_MnO_3_·(1 − x)LiMnO_2_ due to its structural stability. The mechanism still requires further investigation, however, this research provides a novel method to synthesize xLi_2_MnO_3_·(1 − x)LiMnO_2_ cathode material with a low cost and a stable cycling capability for application in lithium-ion batteries.

## 2. Materials and Methods

The samples were prepared via a one-step dynamic hydrothermal method. MnO_2_, Mn(CH_3_COO)_2_·4H_2_O, LiOH·H_2_O, and NaOH were purchased from Aladdin (Shanghai, China). The preparation procedure is described briefly, as follows: 0.04 mol MnO_2_, 0.04 mol Mn(CH_3_COO)_2_·4H_2_O, 0.24 mol LiOH·H_2_O, 0.36 mol NaOH, and 200 mL deionized water were mixed in a 1000 mL dynamic autoclave with a stirrer (stirring rate of 150 rpm). The hydrothermal treatment was carried out at 200 °C for 5 h, with the temperature being increased at a rate of 2 °C min^−1^. Following the reaction, the autoclave was left to cool down to room temperature under ambient conditions. The sample was collected by centrifugation and was washed with deionized water several times, then dried at 80 °C overnight. The final product was marked as LMO-1. LMO-2 and LMO-3 were obtained with the same reagent concentrations, while the volume of solutions were set to 400 and 600 mL of H_2_O, respectively. Furthermore, with the purpose of controlling the atmosphere in the reaction system, the gas in the autoclave was sufficiently purged by compressed air before the hydrothermal process. For use in comparisons, pure o-LiMnO_2_ (marked as LMO-P) was synthesized using ethylene diamine tetraacetic acid disodium salt (EDTA-2Na) according to a previous report [28]. 

The crystal structures were evaluated by powder X-ray diffraction (XRD; X’Pert Pro, PANalytical, Almelo, The Netherlands) with Cu Kα radiation over the range of 2θ = 10–80°. The morphologies of the samples were investigated using scanning electron microscopy (SEM; Nova NanoSEM 450, FEI company, Hillsboro, OR, USA) and transmission electron microscopy (TEM; Tecnai G2F30 S-Twin operated at 300 kV, FEI company, Hillsboro, OR, USA). The size distribution of the samples was analyzed using a particle dimension laser analyzer in dry mode (LS230, Beckman Coulter, Brea, CA, USA). The chemical valence state of the Mn element was confirmed by XPS (Thermo Scientific K-Alpha, Thermo Fisher Scientific, Waltham, MA, USA). The elemental ratio of the samples was analyzed by inductively coupled plasma optical emission spectrometry (ICP–OES, Agilent 720ES, Agilent Technologies, Santa Clara, CA, USA).

The contents of Mn^3+^ and Mn^4+^ from the various samples were obtained via chemical titration according to previous works [29,30]. The final results were the mean values of the triplicate experiments. Chrome blue black R, sulfuric acid, sodium oxalate benchmark solution, EDTA, ammonia–ammonium chloride buffer solution, and ammonium sulfate solution were used in the titration.

The electrochemical performance of the samples was measured using the 2032-type coin cell. A slurry composed of 10 wt.% binder (polyvinylidene fluoride (PVDF)), 10 wt.% conductive additive (Super P Li carbon black), and 80 wt.% active material in N-methyl-2-pyrrolidone (NMP) was cast onto aluminum foil. The electrodes were approximately 100 μm thick and the loading mass of the active material was approximately 1.5 mg cm^−2^. The half-cells were assembled in an argon-filled glovebox employing pure lithium foil as anodes and Celgard 2400 membranes as separators. The electrolyte was composed of a solution of LiPF_6_ (1 M) in ethylene carbonate, ethyl methyl carbonate, and dimethyl carbonate (1:1:1 by volume). The coin cells were galvanostatically cycled on a CT2001A (LAND Electronic Co., Wuhan, China) multi-channel battery test system at room temperature. A cyclic voltammetry (CV) test was conducted on an Ivium electrochemical workstation (Ivium-n-Stat, IVIUM Technologies, Eindhoven, Netherlands) at a scan rate of 0.1 mV s^−1^ between 2.0 and 4.8 V. The electrochemical impedance spectra (EIS) were collected by the Ivium workstation (Ivium-n-Stat, IVIUM Technologies, Eindhoven, Netherlands) within the frequency range of 0.01 Hz–100 kHz with an amplitude of 10 mV.

## 3. Results and Discussion

**Figure 1:**
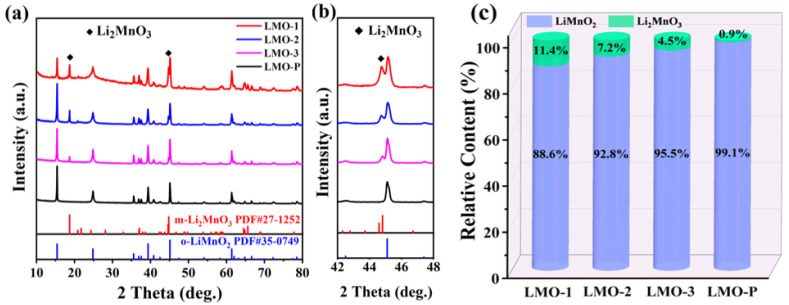
The XRD patterns (**a**,**b**) and ratio of the two phases (**c**) of the four synthesized samples.

The high crystallinity and phase composition of the hydrothermally synthesized xLi_2_MnO_3_·(1 − x)LiMnO_2_ samples were distinctly confirmed by XRD measurement (Figure 1a). The diffraction peaks of LiMnO_2_ can be indexed to the α-NaFeO_2_-type layered structure with a space group of R3m. Meanwhile, the intensity of the peaks at 18.7° and 44.7° can be ascribed to Li_2_MnO_3_ with the C/2m space group, declining gradually as the oxygen in the autoclave decreased, which was more obvious in the enlarged interval between 42° and 48° (Figure 1b). This trend can be attributed to residual oxygen in the reaction system inevitably producing Li_2_MnO_3_, and the chemical reaction can be formulated as follows:

Mn(II) + MnO_2_ + 4Li^+^ + 6OH^−^ + 1/2O_2_ → 2Li_2_MnO_3_ + 3H_2_O

The ICP–OES results in Table 1 present the molar ratio of Li and Mn with the calculation of the Mn valance. The x-values in LMO-1, LMO-2, and LMO-3 are 0.114, 0.072, and 0.045, respectively. Meanwhile, in LMO-P, the content of Li_2_MnO_3_ is only 0.9%, which can be regarded as pure LiMnO_2_. The ratio of the two phases from the different samples is displayed in Figure 1c. In addition, the Mn valence state (Mn^3+^ and Mn^4+^) in each sample was tested using the chemical titration method, and the results are shown in Table 2. According to the chemical titration results, the composition of the xLi_2_MnO_3_·(1 − x)LiMnO_2_ samples is in accordance with the ICP–OES characterization.

**Figure 2:**
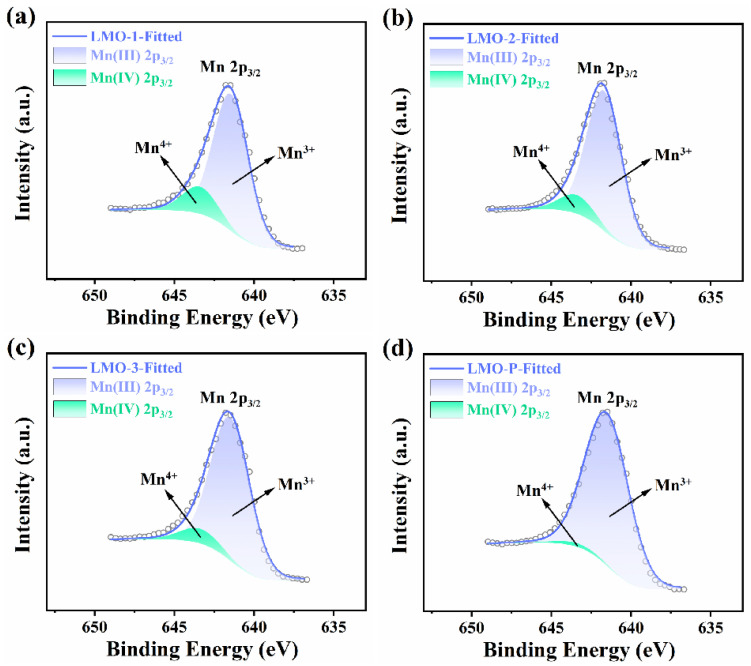
The XPS spectra of the various samples (**a**–**d**).

Figure 2 presents the XPS spectra of four samples, where the chemical state of Mn 2p can be clarified as the combination of Mn^4+^ (2p_3/2_ 643.4 eV) and Mn^3+^ (2p_3/2_ 641.7 eV) [31,32]. The Mn^4+^ content decreased with the decline of oxygen in the reaction system, as reflected by the less Li_2_MnO_3_ in the final product. The area of fitted XPS curves indicates that Mn^3+^ is the majority species, while the Mn in LMO-P sample can be regarded as Mn^3+^ entirely, a conclusion that is consistent with the XRD and ICP results.

**Figure 3:**
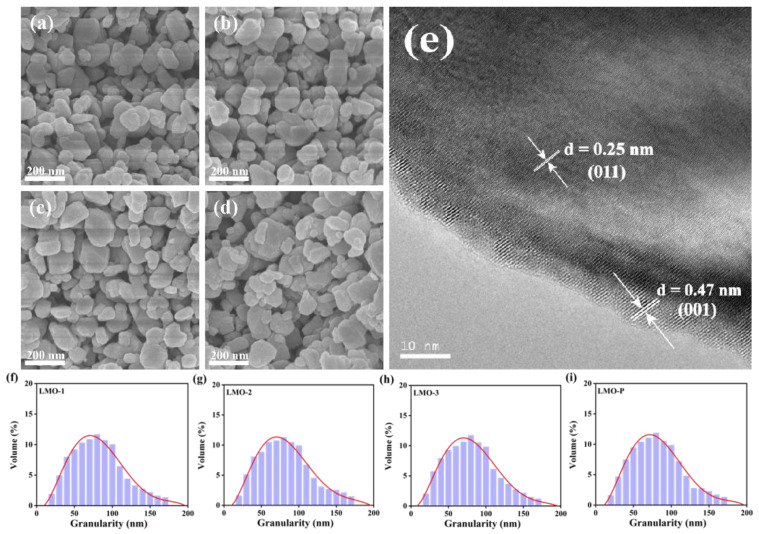
The SEM images of the various samples (**a**–**d**), TEM image of LMO-2 (**e**), and particle size distribution of the samples (**f**–**i**).

The SEM images of four products are presented in Figure 3a–d, all of which show similar morphologies with a particle size ranging from 30 to 150 nm. The size distribution was further examined using a laser particle size analyzer (Figure 3f–i), in which the mean sizes of 81.4, 82.3, 80.9, and 82.1 nm were determined for LMO-1, LMO-2, LMO-3, and LMO-P, respectively. The particle size of the synthesized products is smaller than reported before [11,12]. The use of an alkaline solution with stirring established an appropriate reaction environment for generating nanoparticles with a large surface area and to prevent agglomeration. Hence, the contact area between the active material and electrolyte was enlarged, which produced a shorter diffusion path for Li^+^ to minimize polarization during charging–discharging [33]. 

The HRTEM image of LMO-2 in Figure 3e exhibits lattice fringes with an interplanar space of 0.25 nm, assigned to the (011) crystal plane of orthorhombic LiMnO_2_, and lattice fringes with 0.47 nm of the (001) crystal plane from Li_2_MnO_3_, which confirm the good crystallinity of the product and the co-existence of the two phases. The lattice fringe spacings of LiMnO_2_ (011) and Li_2_MnO_3_ (001) were also calculated based on the XRD pattern of LMO-2. According to the Bragg equation, 2dsinθ = nλ (*n* = 1), the peak at 2θ = 18.7° with a lattice fringe spacing of 4.74Å corresponds to Li_2_MnO_3_ (001), while that at 2θ = 35.6° of 2.52Å is LiMnO_2_ (011), in accordance with HRTEM.

**Figure 4:**
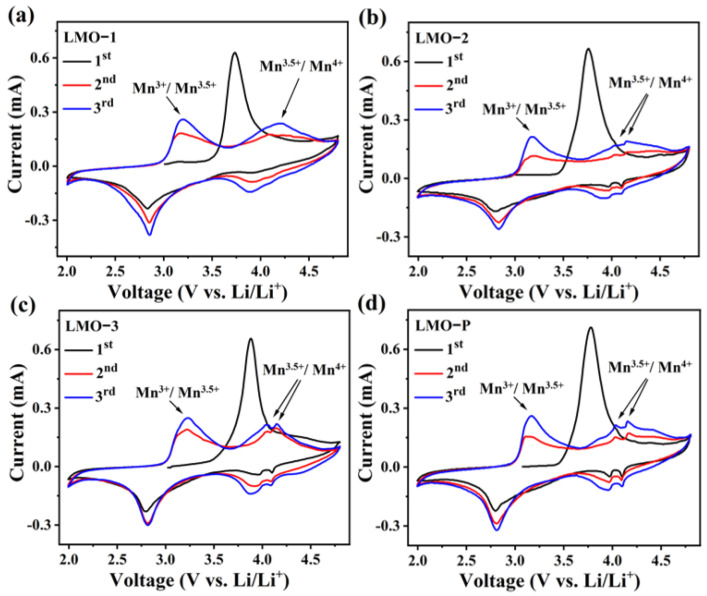
Cyclic voltammograms (CVs) of the various samples in the potential range of 2.0–4.8 V (**a**–**d**).

Figure 4 displays the cyclic voltammograms (CVs) of the various samples in the potential interval from 2.0 to 4.8 V at a scan rate of 0.1 mV s^−1^. The intense oxidation peak at 3.5–4.1 V observed in the first cycle is interpreted as an irreversible de-lithiation from the octahedral sites of LiMnO_2_ upon charging [31,34]. Generally, the products with Li_2_MnO_3_ presented another oxidation peak around 4.7 V due to the activation of Li_2_MnO_3_ with Li^+^ extraction and anion oxidation [34,35]. However, this behavior cannot be distinguished in our research, which demonstrates that the Li_2_MnO_3_ generated via our route is electrochemically inert, even under a high potential. Furthermore, the redox peaks around 3.0 V are ascribed to Mn^3+^/Mn^3.5+^ in Li_2_Mn_2_O_4_ transformed from initial LiMnO_2_ for all of the samples [36]. In the first charging step, this can be explained by the de-lithiation of Li^+^ from octahedral sites and the subsequent migration of Mn^3+^ from the original octahedral sites to neighboring vacant octahedral sites. When Li^+^ intercalates into the de-lithiated matrix in the discharge step, it cannot re-insert into the original octahedral sites, but instead forms tetragonal Li_2_Mn_2_O_4_ [37,38]. Two peaks characteristic of cubic spinel LiMn_2_O_4_ could be detected at 3.95 and 4.10 V for LMO-2, LMO-3, and LMO-P (Figure 4b–d) [39,40]. While for LMO-1 (Figure 4a), these two peaks are merged, this phenomenon should be ascribed to the higher content of inert Li_2_MnO_3_ influencing the lithium ion de-intercalation process in spinel LiMn_2_O_4_ [41]. 

**Figure 5:**
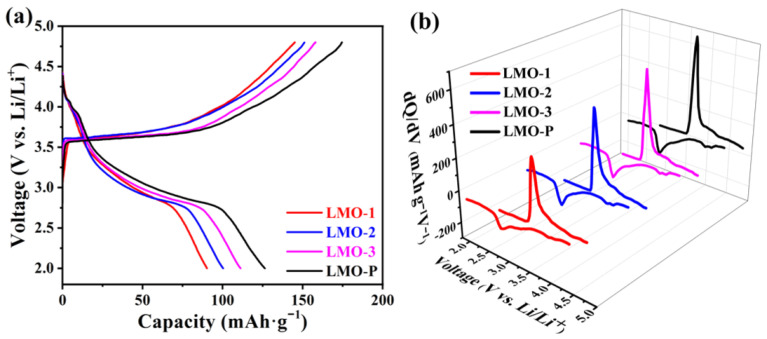
The charge/discharge profiles of the various samples in the first cycle (**a**) and the corresponding dQ/dV curves (**b**).

The charge/discharge profiles of the first cycle are displayed in Figure 5a. The initial charge and discharge plateaus at 3.6 and 3.0 V are characteristic of o-LiMnO_2_, akin to previous reports and in agreement with the results observed by CV [28]. The discharging capacity and coulombic efficiency of the initial cycle is 90.3 mAh g^−1^ (62.2%), 100.3 mAh g^−1^ (66.4%), 111.2 mAh g^−1^ (70.4%), and 126.4 mAh g^−1^ (72.4%) for LMO-1, LMO-2, LMO-3, and LMO-P, respectively. The capacity of each sample is directly proportional to the content of LiMnO_2_, and the coulombic efficiency of the initial cycle declined with the increase in the Li_2_MnO_3_ phase. Such a phenomenon is ascribed to the existence of Li_2_MnO_3_, hindering the de-intercalation of lithium-ion and impeding the phase transformation from LiMnO_2_ to Li_2_Mn_2_O_4_, thus resulting in a low coulombic efficiency. However, once the LiMnO_2_ is entirely transformed to Li_2_Mn_2_O_4_, the existence of Li_2_MnO_3_ was beneficial for preventing structural distortion and improving cycling stability. Furthermore, no obvious peak was present in the related dQ/dV curves in the high potential range of 4.5–4.8 V (Figure 5b). As previously reported, the electrochemical activity of Li_2_MnO_3_ is induced by structural defects, and with more stacking faults, Li_2_MnO_3_ is more easily activated to offer extra capacity, and even the activation the voltage plateau was not obvious [42,43]. However, this is inconsistent with our experimental results, where the highest capacity decreased with the increase in Li_2_MnO_3_ content; thus, the contribution of Li_2_MnO_3_ activation to increasing capacity could be excluded, and the inertness of Li_2_MnO_3_ was confirmed.

**Figure 6:**
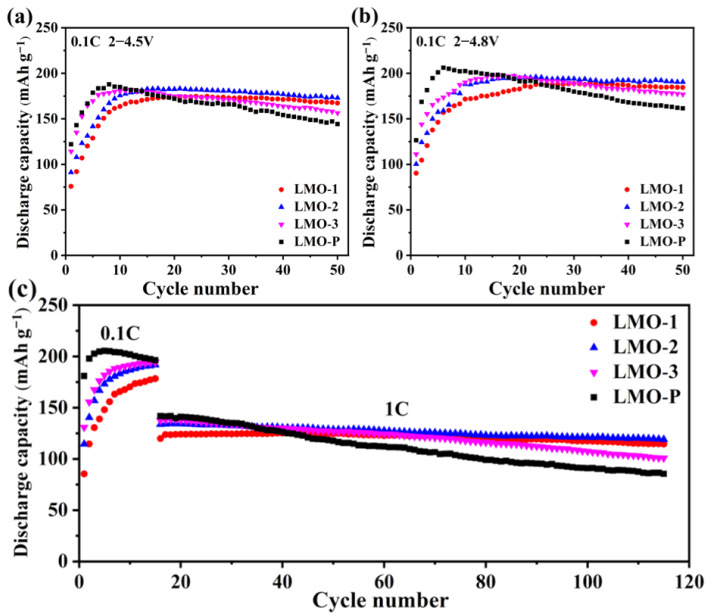
Cycling performance at 0.1 C with the 2.0–4.5 V range (**a**), 0.1 C with the 2.0–4.8 V range (**b**), and 1 C with the 2.0–4.8 V range (**c**).

Plots of the cycling performance of the synthesized materials at 0.1 C (1 C = 300 mA g^−1^) at ranges of 2.0–4.5 and 2.0–4.8 V are shown in Figure 6a,b. Under both conditions, the capacity gradually increased in the initial cycles, which can be attributed to the increasing content of Li_2_Mn_2_O_4_ upon o-LiMnO_2_ transformation [44]. In comparison, the samples cycled with 2.0–4.8 V presented a higher capacity, which is usually explained by the activation of Li_2_MnO_3_ at a high potential to offer extra capacity. Nevertheless, the inertness of Li_2_MnO_3_ in our research implies that more sufficient de-intercalation of Li^+^ at a high potential is responsible for the increase in capacity. This could also explain why the highest capacity decreases with an increase in Li_2_MnO_3_ content.

The speed of the phase transformation from o-LiMnO_2_ to Li_2_Mn_2_O_4_ is related to the content of Li_2_MnO_3_ in the composite [13]. It is clear that LMO-P reached the highest capacity of 206.1 mAh g^−1^ in six cycles with a potential range of 2.0–4.8 V, while the capacity of the 50th cycle was only 161.5 mAh g^−1^ with a retention rate of 78.4%. In comparison, LMO-1, LMO-2, and LMO-3 achieved higher capacities of 189.4, 196.4, and 197.6 mAh g^−1^ in the 33rd, 19th, and 17th cycles, respectively, and the retention rates of the corresponding samples were 97.3%, 97.0%, and 89.5%, respectively. The cycling performance at a rate of 1 C after activation at 0.1 C for 15 cycles is displayed in Figure 6c. The results show that the capacity retention of LMO-P degraded to 60.3%, while LMO-1, LMO-2, and LMO-3 maintained 95.4%, 89.1%, and 73.3%, respectively. The higher charging–discharging rate demands faster Li^+^ de-intercalation and, thus, the structure of a cathode material must be well stabilized, and the existence of Li_2_MnO_3_ effectively improved this factor. The different cycling capability demonstrates that the existence of inert Li_2_MnO_3_ improved the structural stability of the material with deep de-lithiation. Meanwhile, the ratio of Li_2_MnO_3_ in the composite should be optimized to balance the capacity property and the cycling stability, thus achieving the best electrochemical performance; from this point of view, LMO-2 with 7.2% Li_2_MnO_3_ is the most appropriate choice. 

In fact, the capacity degradation associated with LiMnO_2_ is due to Mn dissolution and irreversible layer-to-spinel transformation stemming from Jahn–Teller distortion [40,45]. The introduction of a Li_2_MnO_3_ phase could increase the mean valence of Mn to suppress the disproportionate reaction of Mn^3+^; more importantly, the layered structure is compatible with LiMnO_2_ and the stable layered complex structure can effectively delay irreversible transformation to spinel LiMn_2_O_4_ [13,46].

**Figure 7:**
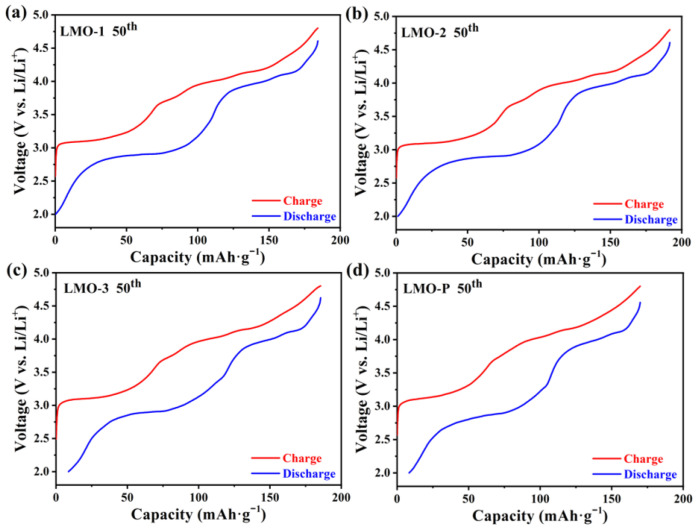
The charging–discharging curves of various samples in the 50th cycle at potential range of 2.0−4.8 V (**a**–**d**).

The charging–discharging curves of different materials in the 50th cycle are shown in Figure 7, in which the voltage hysteresis can be detected. As mentioned earlier, the structure of LiMnO_2_ is transformed and collapses during cycling, and the diffusion path of the lithium-ion then changes to cause voltage hysteresis. In comparison, the existence of Li_2_MnO_3_ relieves voltage hysteresis under either high or low voltage for the redox reaction of Mn^3.5+/4+^ and Mn^3+/3.5+^, which illustrates that Li_2_MnO_3_ suppresses further structural distortion of the material.

**Figure 8:**
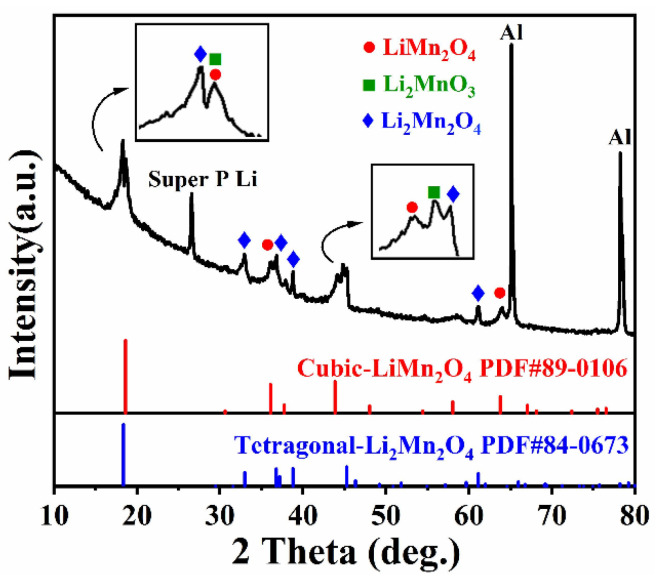
The XRD pattern of the discharged LMO-2 cathode disk after 15 cycles.

In order to identify the structural evolution of the composite, ex situ XRD measurements were taken for the discharged LMO-2 cathode disk after 15 cycles, as shown in Figure 8. The o-LiMnO_2_ phase remarkably vanished, whereas newly formed phases of cubic LiMn_2_O_4_ and tetragonal Li_2_Mn_2_O_4_ could be detected. Moreover, the existence of a Li_2_MnO_3_ phase can be confirmed, which means that Li_2_MnO_3_ did not participate in the electrochemical reaction. The amount of cubic LiMn_2_O_4_ illustrates that inert Li_2_MnO_3_ is not capable of thoroughly suppressing phase transformation, while structure distortion and collapse are prevented, resulting in an improved cycling stability.

**Figure 9:**
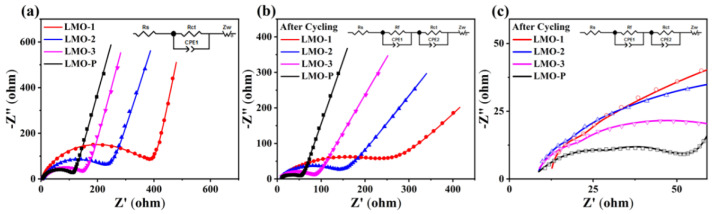
Electrochemical impedance spectra (EIS) of the various samples before (**a**) and after 15 cycles (**b**,**c**).

The electrochemical impedance spectra (EIS) of the samples before and after cycling are shown in Figure 9. The semicircle of a high frequency is ascribed to the surface film resistance (R_f_) formed by the decomposition of the electrolyte, while the semicircle of a high- to medium-frequency represents the charge–transfer resistance (R_ct_) of the electrochemical process, and the line in the low-frequency range indicates a diffusion-controlled process in the solid electrode [47,48]. The fitted values of the simulated circuit displayed in Table 3 demonstrate a decrease in R_ct_ after cycling, which is attributed to the better electronic and ionic conductivity of the transformed LiMn_2_O_4_ phase with a three-dimensional spinel structure [49]. Furthermore, both before and after cycling, the values of R_ct_ were higher for the sample with more Li_2_MnO_3_; this phenomenon implies the inferior conductivity of Li_2_MnO_3_ and that Li_2_MnO_3_ does not change the state even after being cycled under a high potential. On the contrary, R_s_ corresponds to the resistance of the cell, which consists of electrolyte resistance and circuit ohmic resistance. It is supposed that after cycling, the battery is aged, and the electrolyte is partially decomposed to generate impurity, thereby obstructing ion transfer to increase the value of R_s_, as shown in Table 3 [50]. Meanwhile, the formation of an SEI film on the electrode surface blocking the transfer of Li^+^ results in the emergence of R_f_ compared to the EIS before cycling, which is more obvious in the enlarged EIS image after cycling (Figure 9c). 

Compared with the electrochemical properties of the xLi_2_MnO_3_·(1 − x)LiMnO_2_ cathode materials generated using various methods (Table 4), the superiority of those prepared using the dynamic hydrothermal route designed here is remarkable. Our synthetic procedure saves time, and the formation of the 0.072Li_2_MnO_3_·0.928LiMnO_2_ nanocomposite leads to an optimum performance. The specific discharging capacity and cycling stability of the product are much better than the previously reported results, except for 0.23Li_2_MnO_3_·0.77LiMnO_2_ produced via the solid-state method. However, in this composite, Li_2_MnO_3_ is activated after 20 cycles and does not show stability under extended cycling [11]. Modification strategies, including element doping and carbon composition, can also be implemented on the xLi_2_MnO_3_·(1 − x)LiMnO_2_ material to further improve the cycling stability and capacity, which will form the scope of future work.

## 4. Conclusions

A series of xLi_2_MnO_3_·(1 − x)LiMnO_2_ nanocomposites were prepared via a one-step dynamic hydrothermal method. A high concentration of alkaline solution, intense hydrothermal conditions, and stirring were used to produce nanoparticles that possess a large surface area and uniform dispersity. The composite characterized by a good crystallinity was found to be composed of orthorhombic LiMnO_2_ and monoclinic Li_2_MnO_3_. The proportion of the two phases in the composite can be effectively controlled by the amount of oxygen in the autoclave, which, in turn, influences the electrochemical performance.

In contrast with most previously reported lithium-rich materials, the Li_2_MnO_3_ in this composite is completely inert, even when cycled at 2.0–4.8 V, and the typical activation reactions that contribute to extra capacity were not observed. This is ascribed to the high crystallinity and few faults in Li_2_MnO_3_; as an inert phase, Li_2_MnO_3_ effectively suppresses the structural distortion and collapse of the composite during cycling, which results in an improved cycling stability.

The composite with a 0.072Li_2_MnO_3_·0.928LiMnO_2_ composition exhibited the best electrochemical performance and delivered a high capacity of 196.4 mAh g^−^^1^ at 0.1 C under 2–4.8 V. The capacity was maintained at 190.5 mAh g^−^^1^ with a retention rate of 97.0% by the 50^th^ cycle, which demonstrates an excellent cycling stability. This research describes a novel route to synthesize xLi_2_MnO_3_·(1 − x)LiMnO_2_ cathode materials with a low cost and a stable cycling capability, for use in lithium-ion batteries.

## Figures and Tables

**Table 1:** The experimental results of the Li/Mn atom ratio, e Mn average valance, and corresponding calculated x-values.

Sample	Li/Mn _exp_	Li/Mn _theoretical_	Mn Valance _exp_	x Values in xLi_2_MnO_3_·(1 − x)LiMnO_2_
o-LiMnO_2_	1	1	3	0
Li_2_MnO_3_	2	2	4	1
LMO-1	1.114	\	3.114	0.114
LMO-2	1.072	\	3.072	0.072
LMO-3	1.045	\	3.045	0.045
LMO-P	1.009	\	3.009	0.009

**Table 2:** Different manganese valences and their respective content obtained using the chemical titration method, and the composition of xLi_2_MnO_3_·(1 − x)LiMnO_2_.

Sample	Mn^3+^	Mn^4+^	x-Values in xLi_2_MnO_3_·(1 − x)LiMnO_2_
LMO-1	88.8%	11.2%	0.112
LMO-2	92.9%	7.1%	0.071
LMO-3	95.3%	4.7%	0.047
LMO-P	99.3%	0.7%	0.007

**Table 3:** Electrochemical parameters for the alternating current EIS results, calculated using Z-view software.

Samples	As Prepared		After Cycling		
	R_s_ (Ω)	R_ct_ (Ω)	R_s_ (Ω)	R_f_ (Ω)	R_ct_ (Ω)
LMO-1	5.56	384.19	12.71	23.53	237.61
LMO-2	5.88	274.01	8.80	24.88	134.10
LMO-3	5.52	146.73	8.99	27.53	64.07
LMO-P	5.38	112.42	8.75	29.41	30.44

**Table 4:** Summary of the synthetic conditions and electrochemical properties of the obtained lithium manganese oxide cathode materials prepared using different methods.

Product	Method	Voltage Range	Synthesis Condition	Current Density (mA g^−1^)	Maximum/Selected Cycle Discharge Capacity (mAh g^−^^1^)	Reference
0.23Li_2_MnO_3_·0.77LiMnO_2_	Solid state	2.0−4.5 V	750 °C/20 h	20	218/218 (30th)	[11]
0.61Li_2_MnO_3_·0.39LiMnO_2_	Sol–gel	2.0−4.8 V	600 °C/3 h 900 °C/12 h	10	177/167 (30th)	[12]
0.44Li_2_MnO_3_·0.56LiMnO_2_	Hydrothermal + solid state + pyrolysis reduction	2.0−4.8 V	200 °C/2 h 450 °C/10 h 500 °C/15 h 340 °C/4 h	30	270/200 (30th)	[13]
H_0.46_Li_1.54_MnO_3_	Hydrothermal	2.0−4.8 V	180 °C/48 h	200	208/120 (20th)	[14]
LiMnO_2_−Li_2_MnO_3_	Hydrothermal	2.0−4.5 V	200 °C/72 h	10	192/182 (5th)	[51]
o-LiMnO_2_	Hydrothermal	2.0−4.5 V	160 °C/12 h	20	173/162 (20th)	[52]
m-LiMnO_2_ Mixed m/o-LiMnO_2_ o-LiMnO_2_	Hydrothermal	2.0−4.5 V	180 °C/4 h 180 °C/8 h 220 °C/8 h	20	219.8/94.5 (50th) 198.8/112.5 (50th) 180.0/106.8 (50th)	[31]
o-LiMnO_2_ nanorods	Solid state	2.0−4.25 V	750 °C/10 h	20	178.6/165.3 (40th)	[53]
Mesoporous o-LiMnO_2_	Solid state	2.0−4.4 V	600 °C/3 h	20	191.5/162.6 (50th)	[54]
o-LiMnO_2_	Dynamic hydrothermal	2.0−4.5 V	200 °C/3 h	30	166/145 (50th)	[28]
0.072Li_2_MnO_3_·0.928LiMnO_2_	Dynamic hydrothermal	2.0−4.8 V	200 °C/5 h	30	198.4/190.5 (50th)	This work

## Data Availability

The data that support the findings of this study are available from the corresponding author upon reasonable request.
